# Aminodihydrophthalazinedione Sodium Transdermal Therapeutic System Specific Activity on an ExperimentalModel of Extensive Liver Resection

**DOI:** 10.3390/life13030658

**Published:** 2023-02-27

**Authors:** Eugenia G. Kuznetsova, Lydia A. Salomatina, Olga M. Kuryleva, Lyudmila A. Kirsanova, Zalina Z. Gonikova, Alla O. Nikolskaya, Murat Yu. Shagidulin, Natalya P. Shmerko, Victor I. Sevastianov

**Affiliations:** 1 The Shumakov National Medical Research Center of Transplantology and Artificial Organs, 1 Shchukinskaja St., 123182 Moscow, Russia; 2 Autonomous Non-Profit Organization, the Institute of Biomedical Research and Technology, 43/20, Build. 2, B. Tishinsky Lane, 123557 Moscow, Russia

**Keywords:** transdermal therapeutic system, aminodihydrophthalasindione sodium, immunomodulator, extensive liver resection, mitotic index

## Abstract

Previously, the authors showed that the application of the aminodihydrophthalazinedione sodium (ADPS) immunomodulator transdermal therapeutic system (TTS) to laboratory animals provides bioavailability analogous to the intramuscular administration of this drug at the same dose. At the same time, its maximum blood concentration is significantly reduced, and the retention time of the drug in the body is increased more than 10-fold, which can contribute to prolonging the drug effect. The aim of the work was to identify a possible positive effect of the transdermal administration of the ADPS immunomodulator on reparative liver regeneration on an experimental model of extensive liver resection (ELR). It has been shown that at a period of 48 h after ELR, the percutaneous administration of the immunomodulator has a pronounced stimulating effect on the mitotic activity of rat liver cells; by 72 h after ELR, an accelerated rate of recovery of hepatic homeostasis in the body was observed in laboratory animals in groups with the application of the ADPS TTS versus the control group.

## 1. Introduction

Over the past decades, there has been a surge in scientific research in the development and implementation of highly active and competitive dosage forms in medical practice. Increasing attention is being paid to new drug delivery systems with improved biopharmaceutical characteristics that boost the therapeutic efficacy, tolerability, and safety of drug therapy. One such direction is the creation of transdermal therapeutic systems (TTS)—controlled release dosage forms designed for the continuous supply of drugs through intact skin into systemic circulation for a long time, limited only by medical indications. The use of a transdermal therapeutic system not only allows for increasing the drugs’ bioavailability but also eliminates the disadvantages of other methods of their administration [1,2,3]. In recent years, numerous new drug introductions have been observed, which can be delivered in a transdermal manner [4]. This is possible with new developments in the field of methods of skin penetration of medicinal substances. In particular, transdermal drug delivery systems have proved their potential to cure cancer. They increase the bioavailability of the drug by site-specific drug delivery and can reduce side effects and toxicity associated with anticancer drugs [5]. In addition, transdermal delivery has emerged as an appealing approach to enable high efficiency and low toxicity [6].

One of the prospective Russian-made drugs with an immunomodulatory property as well as an expressed anti-inflammatory property is a synthetic low-molecular-weight medicinal substance, aminodihydrophthalasindione sodium (ADPS), a derivative of phthalhydroside. The mechanism of action, in this case, is associated with its ability to reversibly (for 6–8 h) inhibit the excessive synthesis of tumor necrosis factor-α, interleukin-1, interleukin-6, and other proinflammatory cytokines by hyperactivated macrophages. In addition, ADPS stimulates the production of macrophages of key growth factors regulating regenerative processes, angiogenesis, and collagen production in the focus of inflammation [7].

The authors have developed a transdermal therapeutic system containing the ADPS synthetic immunomodulator [8]. Such a dosage form will make it possible to use this drug in various groups of patients who, for several reasons and/or contraindications, cannot use the existing dosage forms of ADPS.

It was shown in vivo that the bioavailability of the transdermal administration of an immunomodulator is equal to the bioavailability of the intramuscular administration of this medicinal substance at the same dose. At the same time, the maximum blood concentration of ADPS is significantly reduced, but the retention time of this immunomodulator in the body is increased by more than 10-fold, which may contribute to the prolongation of the medicinal effect [9]. However, the percutaneous transfer of a medicinal substance is a necessary but insufficient condition for its specific activity.

There are data on the positive effect of various immunomodulators by injectable administration on the process of liver tissue repair in experiments [10,11].

To confirm the specific activity of the ADPS immunomodulator transdermal administration, the authors used a model of extensive liver resection (ELR) in rats.

Most knowledge about liver regeneration is obtained from rodent models where a two-thirds partial hepatectomy was performed [12]. This type of surgery is related to a critical injury since 60% or more of the total mass of the organ is removed [13]. As a result, clinical manifestations of acute liver failure often occur in the postoperative period, while the surgically removed volume of the liver is restored within one week due to internal reparative regeneration [12].

Liver regeneration after partial hepatectomy is carried out with the participation of all mature types of liver cells. The liver manages to restore any lost mass and adjust its size to the size of the body while at the same time providing full support for the body’s homeostasis throughout the regeneration process [14,15]. At the same time, antiproliferative factors control the rate of proliferation and determine the endpoint of liver regeneration. In addition, antiproliferative factors steer the regeneration process in the right direction, preventing the pathological reproduction of abnormal cells, as it occurs during oncogenesis. Therefore, proliferation inhibitors are just as important for ensuring safe and stable liver regeneration as the factors that promote proliferation [16]. These processes are controlled by cytokines, growth factors, and signaling pathways. Cytokines, including transforming growth factor β and interleukin-1, and tumor suppressor genes are important members of the family of proliferation inhibitors during liver regeneration [14].

In clinical practice, the problem of accelerated liver recovery after extensive resection in cancer patients, as well as in donors with related transplantation of the right lobe of the liver, is of the greatest interest [17].

It is worth noting that an important role in the regulation of regenerative processes in the body belongs to the immune system, all elements of which are actively involved in restoring the structure and function of cells of the damaged tissue. In this regard, the question arises about the expediency of using immunological drugs to impact the reparative processes in the affected organs and tissues [10,18]. The scientific literature describes studies of the positive effect of various immunosuppressants on liver regeneration. In particular, Giorgio Alfredo Pedroso Baretta et al. describe the positive effect of cyclosporine on liver regeneration in rats undergoing 70% hepatectomy in an experimental model [19]. The positive effect of tacrolimus-based immunosuppressive therapy in adult Wistar rats on a similar experimental model is also described. Its stimulating effect on liver regeneration is shown [20]. There is also information about the partial half-life effect of sirolimus on liver regeneration caused by the resection of 70% of the liver of adult rats [21].

It is known that the mitotic activity of hepatocytes is reduced on the first day after surgery, but already on the second day its increase is noted [22]. The maximum mitotic and functional activity of hepatocytes is observed between the second and fifth days after resection [22,23]. It can be assumed that the use of a prolonged transdermal dosage form will enhance the natural process of liver regeneration by maintaining a constant blood concentration of an immunomodulator for the required time period.

The aim of the work was to identify a possible positive effect of the transdermal administration of the *ADPS* immunomodulator on reparative liver regeneration in an experimental model of extensive liver resection.

## 2. Materials and Methods

### 2.1. Materials for the Production of Laboratory Samples of the Immunomodulator Transdermal Therapeutic System

Deionized water (Simplicity^®^ UV, Millipore, NJ, USA), 0.9% sodium chloride solution (ESCOM, Stavropol, Russia), sodium dodecyl sulfate (C_12_H_25_SO_4_Na, AppliChem Panreac, Barcelona, Spain), apricot kernel oil (oil with a similar fatty profile—3 to 10% palmitic acid (C_16_H_23_O_2_), 0.5–4% stearic acid (C_17_H_35_COOH), 55 to 70% oleic acid (C_18_H_34_O_2_), and 22 to 35% linoleic acid (C_18_H_32_O_2_), max 1% linolenic acid (C_18_H_32_O_2_) (Desert Whale Jojoba Company Ltd., Tucson, AZ, USA), α-tocopherol acetate (C_31_H_52_O, BASF SE, Ludwigshafen am Rhein, Germany), sodium docusate (C_20_H_37_NaO_7_S, Sigma, Saint Louis, MO, USA), Decaglyn PR-20 emulsifier ((C3H5O_2_)_n_(C_18_H_32_O_2_)_m_, Nikko Chemicals Co., Ltd., Tokyo, Japan) were used in the manufacturing of matrix-type laboratory samples of the aminodihydrophthalasindione sodium transdermal therapeutic system (“SELVIM”, Belgorod, Russia) with an emulsion delivery system [8]. As auxiliary materials of the TTS, the following were selected: elastic micro-sponge Foam tape 9773 (3M, Saint Paul, MN, USA), sorbing base PALV-01 (Palm Group of Companies LLC, Moscow, Russia), and Skotchpak 9730 film (3M, Saint Paul, USA). Equipment: dispersant (Heidolph DIAX 900, Schwabach, Germany), ultrasonic homogenizer (Heilscher UIS250V, Teltow, Germany), analytical scales (GH-200 AND, Tokyo, Japan), centrifuge (Hettich Rotina 38R, Kirchlengern, Germany).

### 2.2. The Production of Laboratory Samples of the Immunomodulator Transdermal Therapeutic System

The ADPS transdermal therapeutic system consists of an outer cover layer, a sorbing base with an emulsion composition containing a medicinal substance and enhancers, and an anti-adhesive film removed before application.

To create an emulsion composition oil phase with a medicinal substance, the required amount of a 10% solution of α-tocopherol acetate in apricot kernel oil was heated to 60 °C, and then a lipophilic emulsifier and an enhancer sodium docusate were added. All components were mixed. An aqueous solution with a medicinal substance was added to the heated oil phase of the emulsion composition and mixed until an emulsion was formed. Then the emulsion composition was exposed to an ultrasonic homogenizer for a certain time.

**Figure 1:**
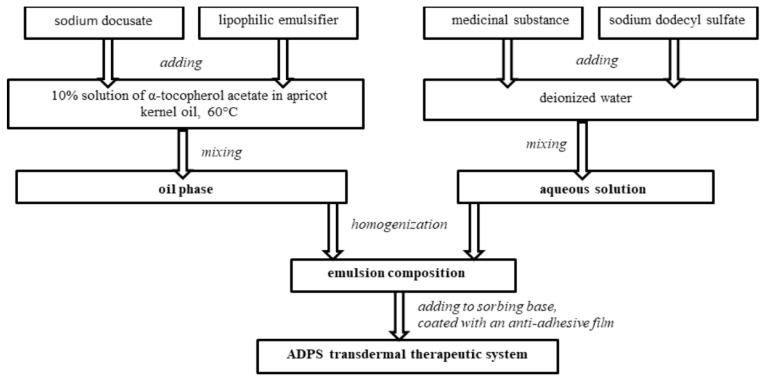
Diagram of preparing an ADPS transdermal therapeutic system.

The emulsion composition was added to a 10 cm^2^-sorbing base, controlling the weight on the scales. Each TTS contains 40 mg of aminodihydrophthalasindione sodium, which corresponds to the ADPS human equivalent dose for rats. Laboratory samples of the ADPS TTS were covered with an anti-adhesive film (Figure 1).

An ADPS TTS has the capacity for prolonged storage with the preservation of pharmacological activity for 1 year during storage with the observance of temperature modes (not lower than +5 and not higher than +25 °C). The stability of laboratory samples was assessed on the basis of a drug content test by high-performance liquid chromatography.

### 2.3. Experimental Model of Extensive Liver Resection

Wistar rats (males) weighing 350–380 g (n = 47) were used in experiments. Manipulations did not cause pain to the animals and were carried out in compliance with Russian legislation: GOST 33215-2014 (Guidelines for accommodation and care of laboratory animals. Rules for the equipment of premises and organization of procedures) and GOST 33216-2014 (Guidelines for accommodation and care of laboratory animals. Rules for the accommodation and care of laboratory rodents and rabbits). The work was approved by the Local Ethics Committee at the Shumakov National Medical Research Center of Transplantology and Artificial Organs, Moscow, Russia (28 January 2021, Protocol No. 280121-1/1e).

**Figure 2:**
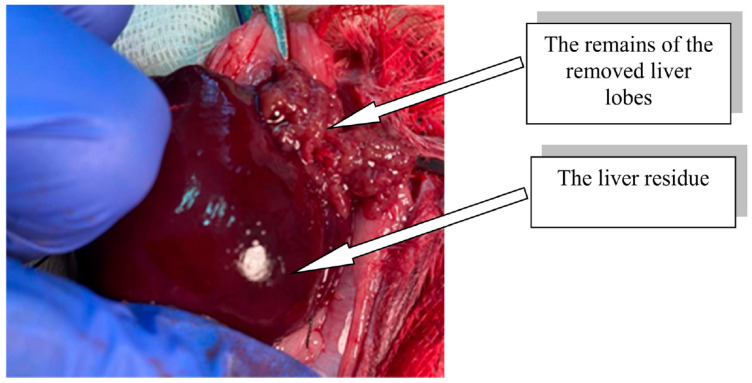
Rat liver residue after extensive resection.

The modeling of extensive liver resection in rats was performed under anesthesia (Zoletil 100, Virbac, Carros, France, 15 mg/kg). In compliance with the rules of asepsis and antiseptics, the abdominal cavity was opened, the liver was moved into the wound, and ligatures were sequentially applied to the bases of the median, left lateral, and right upper lobes of the liver, after which ~70% of the total liver mass was removed (Figure 2) [24].

It is known that the degree and timing of liver regeneration vary depending on circadian rhythms [25]. The operation was always performed in the morning (in the period from 10 to 12 a.m.) when the daily rhythm of the mitotic activity of liver cells is minimal [26]. Note that in all groups of animals, lethality was absent during the entire duration of the experiment.

### 2.4. The Research Design

All the animals were divided into seven groups: six groups of rats (n = 42) were used to create the model of ELR, and a group of intact rats (n = 5). Animal observations were carried out during the periods of 24, 48, and 72 h after ELR. The periods of 48 and 72 h were selected because the maximum mitotic and functional activity of hepatocytes is observed between the second and fifth days after ELR [22,23]. A period of 24 h was selected to detect the impact of the ADPS TTS application on the earlier functional activity of hepatocytes. At each follow-up period, there were two groups of animals with ELR: with the application of an immunomodulator TTS and without the use of an immunomodulator TTS (control groups) (Table 1).

**Figure 3:**
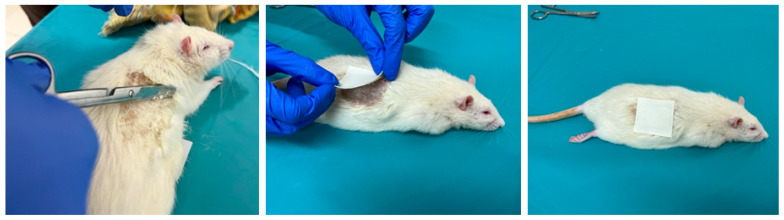
The process of preparing the skin of a laboratory animal and the application of ADPS TTS.

Animals were treated with the ADPS TTS on the dorsal skin area with previously removed hair immediately after liver resection in groups 24/1, 48/1, and 72/1 (Figure 3).

The duration of application was 24 h in groups 24/1 and 24/2; in groups 48/1 and 48/2—48 h with a single replacement of TTS after 24 h; in groups 72/1 and 72/2—72 h with a double replacement of TTS every 24 h.

### 2.5. Study of Biochemical Parameters of the Blood of Laboratory Animals

The following biochemical parameters were determined on the Reflotron™ analyzer (Roche Diagnostics, Rotkreuz, Switzerland): alanine aminotransferase (ALT), aspartic aminotransferase (AST), gammaglutamintranspeptidase (GGT), alkaline phosphatase (ALP), total protein, albumin, total bilirubin, creatinine, and urea.

To accomplish this, the tip of a rat’s tail was dissected under anesthesia (Zoletil 100, Virbac, Carros, France, 5 mg/kg), and blood (32 mL) was taken with a pipette and applied to Reflotron™ test strips, which were immediately placed in a biochemical analyzer.

### 2.6. Study of the Recovery Dynamics of the Laboratory Animals Liver Mass

To assess liver mass recovery dynamics in each operated animal, the removed part of the liver was weighed on an electronic scale (Ohaus Explorer, Nanikon, Switzerland) immediately after ELR. This removed part of the liver was taken as 70% of the total liver mass. Then, based on these data, the initial mass of the liver remainder for each animal was calculated. Then, after 24, 48, or 72 h of TTS application (in accordance with the labeling of the group), the remaining part of the liver was excised, its mass was measured, and the values obtained were compared with the calculated initial mass of the liver remainder for this laboratory animal.

### 2.7. Histological Staining

The effectiveness of the stimulating effect of the percutaneous administration of an immunomodulator on the processes of liver regeneration after ELR was evaluated by the mitotic (proliferative) activity of hepatocytes in the remainder of the resected liver. To do this, the extracted fragment of the middle lobe of the liver was cut into 3 × 4 × 5 mm pieces and placed in Buena solution for fixation. After 24 h, the Buena solution was replaced with 70% ethyl alcohol. When the fixation was completed, the pieces were washed in running water for 2–3 h, dehydrated in alcohols of ascending concentration, and poured into paraffin. Paraffin sections with a thickness of 4–5 µk were wet-glued onto glasses with a poly-L-lysine coating (Thermo, Waltham, MA, USA). Then the preparations were dried for 48 h in a thermostat at 37 °C, dewaxed, rehydrated, and stained with hematoxylin and eosin.

### 2.8. Assessment of the Mitotic Index of the Laboratory Animals Liver

The mitotic activity of hepatocytes was studied after staining histological preparations of the excised liver. For each sample, the number of mitosis figures and the total number of cells were determined on a histological section of liver tissue stained with hematoxylin and eosin with an ×200 microscope magnification (Leica DM LS, Wetzlar, Germany). This formula was used for the mitotic index calculation (*MI*, ‰):

$$\mathrm{MI}=\frac{M}{N}1000$$

*M*—the number of dividing cells,
*N*—the total number of analyzed cells.

### 2.9. Statistical Analysis

Using the Shapiro-Wilkes test on a small number of samples (n ≥ 5), a regular distribution of experimental data was proved. The validity of differences was determined using Student’s *t*-test (standard software package Microsoft Excel 2007). The differences were considered statistically significant if *p* < 0.05.

## 3. Results

### 3.1. Assessment of the Mitotic Index

**Figure 4:**
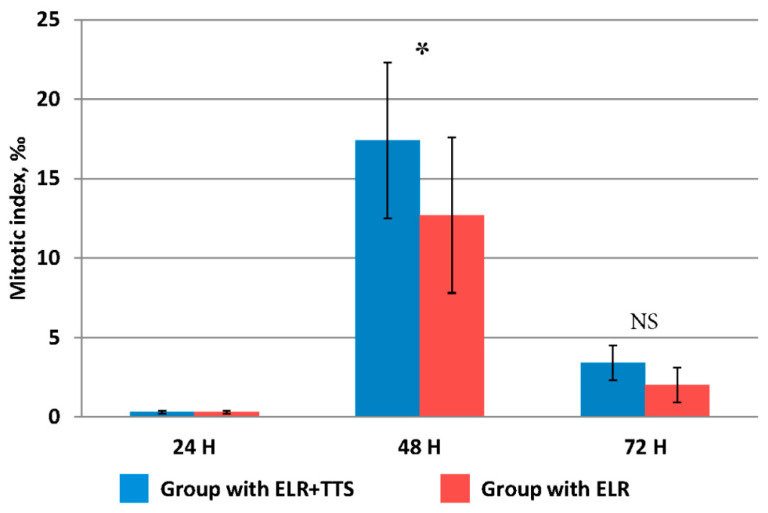
Change in the value of the mitotic index of hepatocytes after ELR, * *p* ≤ 0.05, NS *p* > 0.05.

It is known that after extensive liver resection, the organ is restored to its original mass due to the proliferation and polyploidization of hepatocytes. It was noted that the distinct signs of increased proliferative activity of liver cells after extensive resection appears only after 48 h [27]. However, taking into account the possible stimulation of this process by the percutaneous administration of the ADPS immunomodulator, the evaluation of the proliferative activity of hepatocytes in the remaining part of the resected liver was carried out at periods of 24, 48, and 72 h from the start of the TTS application (Figure 4).

The mitotic activity of hepatocytes at 24 h after ELR in both groups did not differ from the initial value immediately after resection, equaling 0.14 ± 0.07‰. At 48 h after ELR, a significant increase was established, and in the group with TTS application, the mitotic index was significantly higher than in that without it (*p* ≤ 0.05). At 72 h after ELR, the mitotic activity of hepatocytes decreased fourfold, while its values did not differ in both groups (*p* > 0.05).

Thus, at 48 h after ELR, the percutaneous administration of the ADPS immunomodulator had a pronounced stimulating effect on the mitotic activity of liver cells.

**Figure 5:**
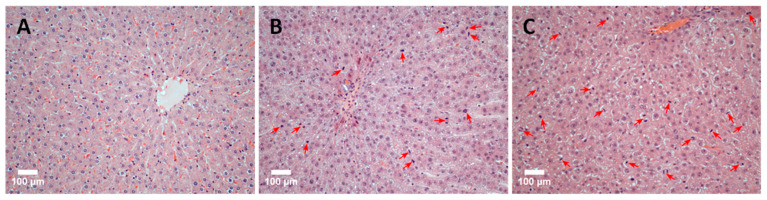
A histological picture of the rat liver tissue (scale bar = 100 µm). The original rat liver tissue: a single instance of mitosis in the parenchyma indicated by an arrow (**A**). Mitotic activity of hepatocytes 48 h after ELR: arrows indicate hepatocytes in the stage of mitosis (**B**). Mitotic activity of hepatocytes 48 h after ELR and the immunomodulator TTS application: numerous mitotic figures in the field of view indicated by arrows (**C**).

The discovered patterns are confirmed by the results of a comparative histological analysis of liver tissue sections. Figure 5 shows photos of histological preparations of rat liver tissue: immediately after ELR (normal tissue), 48 h after ELR, and the application of the immunomodulator TTS (group 48/1) and 48 h after ELR (group 48/2). The images illustrate the results well: in comparison with normal tissue (Figure 5A), the high mitotic activity of hepatocytes is noticeable in group 48/2 (Figure 5B). An even more pronounced manifestation of mitotic activity of hepatocytes is noted in the 48/1 group with the application of the ADPS TTS (Figure 5C).

### 3.2. Results of the Study of Liver Remainder Weight Increase

It should be noted that there was no significant difference in the weight gain of the resected liver in experimental animals in the group with the application of the immunomodulator TTS and the control group at any of the follow-up periods.

### 3.3. Research of Blood Biochemical Parameters

It has been established that the main predictors of hepatic dysfunction are the levels of total bilirubin, albumin, creatinine, alanine aminotransferase, aspartate aminotransferase, and gammaglutamintranspeptidase [28]. In addition to the above blood biochemical parameters during liver resection, changes such as alkaline phosphatase, urea, and total protein should be taken into account [29].

**Figure 6:**
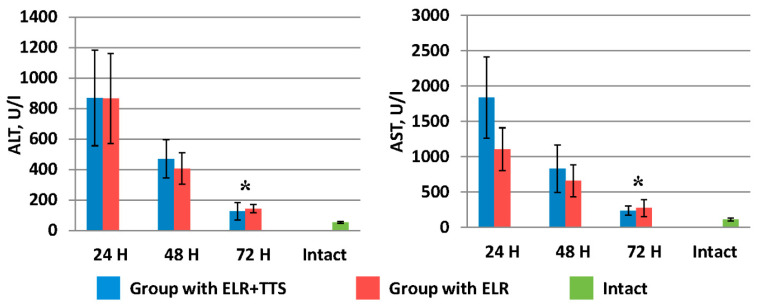
Dynamics of the decrease in liver transaminases after ELR in the blood of laboratory animals, * *p* < 0.05.

The effect of the ADPS immunomodulator on the processes of restoring hepatic homeostasis in rats was studied at 24, 48, and 72 h after ELR (Figure 3, Figure 4, Figure 5 and Figure 6). As can be seen from the figures, 24 h after ELR, changes in the biochemical parameters of the blood of animals in both groups are noted versus the intact group of laboratory animals, which is a predictable reaction of the body after liver resection [29].

By 72 h after ELR, an accelerated rate of recovery of hepatic homeostasis in the body was observed in laboratory animals in groups with the application of ADPS.

Figure 6 shows the dynamics of the decrease in liver transaminases (ALT, AST) after ELR in the blood of laboratory animals. It should be noted that by 72 h in the group of animals with the ADPS TTS application, the ALT and AST values were 126.0 ± 58.0 U/I and 238.5 ± 66.0 U/I, respectively, which is significantly lower (*p* < 0.05) than similar indicators in the group without TTS application (ALT 142.0 ± 26.8 U/I and AST 273.1 ± 119.4 U/I).

**Figure 7:**
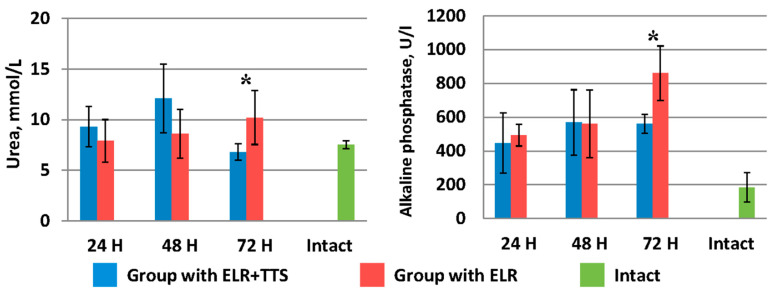
Dynamics of urea and alkaline phosphatase after ELR in the blood of laboratory animals, * *p* < 0.05.

As can be seen from Figure 7, in the group of laboratory animals without TTS, blood urea levels increase from 7.9 ± 2.1 mmol/L (24 h after ELR) to 10.20 ± 2.66 mmol/L (72 h after ELR). A similar pattern is observed for alkaline phosphatase indices (24 h after ELR 493.0 ± 63.9 U/I, 72 h after ELR 860.0 ± 161.2 U/I).

In the group of laboratory animals with the application of ADPS TTS, there was an increase in the level of urea and alkaline phosphatase from 24 to 48 h of the experiment. However, by 72 h of observation, there is a sharp decrease in the urea index to 6.8 ± 0.8 mmol/L, which corresponds to normal values (in the intact group—7.5 ± 0.4 mmol/L). In the period from 48 to 72 h of observation, the alkaline phosphatase index remained at the same level (⁓560 mmol/L), which is significantly lower (*p* < 0.05) than this indicator in the control group of animals.

**Figure 8:**
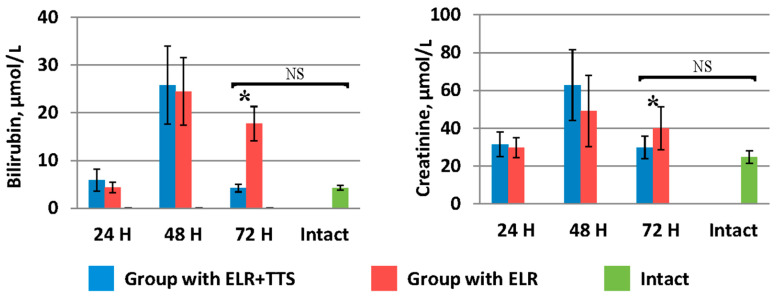
Dynamics of bilirubin and creatinine after ELR in the blood of laboratory animals, * *p* < 0.05, NS *p* > 0.05.

Indicators of bilirubin and creatinine in the blood (Figure 8) in the group of laboratory animals with TTS application by 72 h after ELR (4.2 ± 0.8 µmol/L and 29.8 ± 5.9 µmol/L, respectively) were significantly lower (*p* < 0.05) than in the control group (17.7 ± 3.6 µmol/L and 39.90 ± 11.34 µmol/L, respectively), and did not significantly differ (*p* > 0.05) from the values of the corresponding biochemical parameters of the intact group (4.25 ± 0.5 µmol/L and 24.7 ± 3.3 µmol/L, respectively).

**Figure 9:**
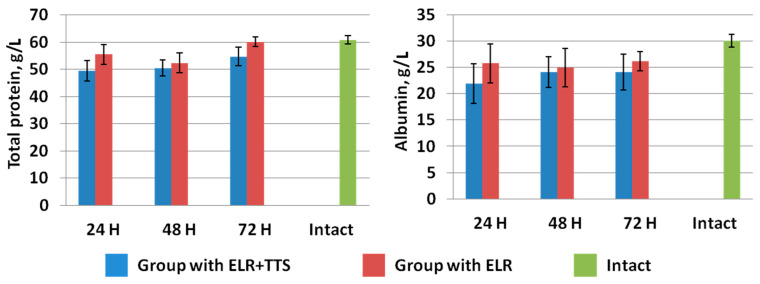
Dynamics of total protein and albumin after ELR in the blood of laboratory animals.

In the study of total protein and albumin in the blood of laboratory animals, it was found that a day after ELR, a gradual increase in these indicators was observed in both groups (Figure 9). At the same time, in the group of animals with the application of ADPS TTS, there is a slowdown in the growth of the level of total protein and albumin versus the control group of operated animals. Note that the intramuscular administration of ADPS, on the contrary, causes an increase in proteinemia [30]. It can be assumed that in the case of a transdermal administration, biotransformation of the molecules of this medicinal substance by the enzymes of the epidermis and dermis occurs, which can lead to a partial loss of the effectiveness of the drug [31]. To explain the results obtained, it is planned to conduct additional studies of other indicators of protein metabolism, such as colloidal-oncotic pressure and the content of prealbumin and globulins.

Evaluating the results of the effect of a percutaneous administration of an immunomodulator on hepatic homeostasis restoration, it can be concluded that a faster normalization of the majority of biochemical blood parameters is observed in the group of laboratory animals with the application of the ADPS TTS.

## 4. Discussion

In recent years, the range of medicinal substances used in the form of transdermal therapeutic systems has been significantly expanding. This is due not only to a number of advantages of the percutaneous administration of medicinal substances in comparison with traditional methods but also to the improvement of methods of influencing the percutaneous transport of medicinal molecules [32,33]. Currently, TTS are appearing on the market, containing, for example, medicinal substances for gene therapy, for the treatment of dementia in geriatric practice, vaccines, hormones, etc. [4]. This method of introducing drugs into the body is characterized by simplicity and ease of use. We have not found publications on the use of a transdermal method of administration of liver regeneration stimulants in the literature.

The relevance of studies related to the search for stimulators of liver regeneration is due to the need to increase the effectiveness of rehabilitation therapy to prevent the development of liver failure in patients after extensive liver resection and donors with related transplants [28].

It is known that during the surgical intervention in the hepatic parenchyma, along with the death of liver cells, their regeneration processes occur simultaneously, and under physiological conditions, the regeneration of hepatocytes proceeds much faster than their death. However, despite the fact that no organ can compare with the liver in terms of the intensity of this process, its physiological regeneration is still very slow [28]. Our studies have shown that the percutaneous administration of the ADPS immunomodulator can affect the reparative processes in the liver tissue of laboratory animals after ELR.

There are works on the use of various ways to stimulate liver regenerative processes: the introduction of various pharmaceutical and biological agents or products of cellular technologies.

A number of works are devoted to the study of the effect of nicotinamide on liver regeneration and the restoration of its function. For example, in tests conducted on a liver resection model in C57BL/6 mice using immunohistochemical analysis (study of the Ki-67 monoclonal antibody), it was shown that the addition of exogenous nicotinamide (Nam) promotes the proliferation of hepatocytes and accelerates the recovery of liver tissue. The addition of Nam protected liver function after resection, which is confirmed by the data from studying the morphology of liver tissue and changes in serum markers of its damage [34]. It was observed that the serum levels of ALT and AST were significantly decreased following ELR in the treatment group compared with the control group. Similar results were obtained in other studies [35]. In our work, with the simultaneous ADPS immunomodulator percutaneous administration, a decrease in ALT and AST levels is also observed. It should be noted that when applying the ADPS TTC 72 h after ELR, the level of these indicators approximately corresponds to the values of serum markers with the intraperitoneal administration of Nam.

Changes in the same parameters showing the deterioration of liver cells after resection was observed by O. Parlak et al. when evaluating the L-carnitine effect on liver regeneration in an experiment [36]. On a liver resection model in Wistar albino rats, an improvement in hepatic homeostasis was achieved by the parenteral administration of L-carnitine at a dose of 100 mg/kg/day. In the same work, an increase in the number of mitoses and the hepatocyte proliferation index under the action of this drug was proved. Note that in our work, a significant increase in the number of mitoses was caused by the application of ADPS TTS compared to the control group of animals.

C. Mangieri et al. carried out the assessment of the ongoing liver regeneration in rats with an intraoperative infusion of hepatocyte growth factor (HGF). It was found that this type of HGF infusion significantly increases liver regeneration after portal branch ligation in rodents [37].

Experimental studies on mice were also described, where the role of bile salts in liver regeneration after its resection was shown. Under the influence of these salts, after hepatectomy, bile salt receptors are activated in non-parenchymatous (plasma membrane receptor TGR5) and parenchymal (intracellular receptor Fxr) liver cells, thereby providing signals for regenerative processes [38].

Work is also underway to study MicroRNAs (miRNAs) as stimulators of liver regeneration [13,39]. For example, G. Song et al. studied the effect of miRNAs on the regulation of hepatocyte proliferation during liver regeneration in experiments on mice. Studies have shown that miRNA are critical regulators of hepatocyte proliferation during liver regeneration [40].

It should be noted that all these works are still at the stage of fundamental research and require a further detailed study for clinical practice applications. The above methods of stimulating liver reparative processes using cellular technologies are more complex, time-consuming, and expensive compared to the ADPS TTS developed by the authors. The ADPS TTS developed by us is a three-layer patch. The first layer is a backing film used as the outermost layer of a transdermal system to protect the product during wear, the second line is a nonwoven material containing an emulsion composition with an immunomodulator and percutaneous carriers, and the third layer is an anti-adhesive film that is removed before use. The composition of the emulsion was published earlier [8].

## 5. Conclusions

The studies presented in this article are aimed at proving the possibility of using a prolonged form of an immunomodulator as a maintenance therapy to stimulate the regeneration of a damaged liver.

At the first stage, a new dosage form of the immunomodulator was developed—the ADPS TTS, with the presence of percutaneous transfer of the drug substance proved in vitro [8]. In the second stage, the pharmacokinetic studies of the immunomodulator TTS were carried out [9]. It has been shown that the transdermal delivery of ADPS, compared with its intramuscular administration, increases the average retention time of the drug in the body more than 12–15 fold. The half-life is also increased more than 10 -old. However, the presence of a transdermal drug transfer is a necessary but not sufficient condition for its specific activity. In this regard, the next stage was the identification of a possible positive effect of the ADPS immunomodulator transdermal administration on reparative liver regeneration in an experimental model of extensive liver resection.

The results of studying the mitotic activity of hepatocytes in the early stages following extensive liver resection in the studied groups of animals showed that ELR induces the proliferative activity of hepatocytes, while percutaneous administration of the ADPS immunomodulator after ELR has a positive effect on the proliferation of hepatocytes in the remainder of the resected liver. It should be noted that on the third day after ELR, with the percutaneous administration of ADPS, there was a tendency to a faster normalization of the blood biochemical parameters of experimental animals versus the control group of operated animals (without the application of TTS). It should be emphasized that the stimulating effect of the ADPS immunomodulator on the regeneration of the damaged liver was established for rats with a high level of internal reparative liver regeneration.

The revealed inhibitory effect of the percutaneous administration of ADPS on the normalization of the level of total protein and albumin in blood after ELR requires additional studies.

Note that in the future, we aim to compare the effectiveness of oral, injectable, and transdermal methods of administration. These studies are planned in the ADPS TTS Preclinical Research Program.

## Figures and Tables

**Table 1:** The list of experimental groups of laboratory animals.

Marking of the Group (Study Duration, h/Group Number)	Groups	Number of Animals
24/1	Laboratory animals after ELR and TTS application within 24 h	5
24/2	Laboratory animals after ELR (after 24 h) without TTS application	5
48/1	Laboratory animals after ELR and TTS application within 48 h	12
48/2	Laboratory animals after ELR (after 48 h) without TTS application	10
72/1	Laboratory animals after ELR and TTS application within 72 h	5
72/2	Laboratory animals after ELR (after 72 h) without TTS application	5
	Intact animals	5

## Data Availability

Not applicable.
